# Bortezomib-mediated down-regulation of telomerase and disruption of telomere homeostasis contributes to apoptosis of malignant cells

**DOI:** 10.18632/oncotarget.5752

**Published:** 2015-10-12

**Authors:** Xinyu Ci, Bingnan Li, Xueping Ma, Feng Kong, Chengyun Zheng, Magnus Björkholm, Jihui Jia, Dawei Xu

**Affiliations:** ^1^ Central Research Laboratory, Shandong University Second Hospital, Jinan, PR China; ^2^ Department of Microbiology, School of Medicine, Shandong University, Jinan, PR China; ^3^ Division of Hematology, Department of Medicine and Center for Molecular Medicine, Karolinska Institutet and Karolinska University Hospital Solna, Stockholm, Sweden; ^4^ Karolinska Institutet-Shandong University Collaborative Laboratories for Cancer and Stem Cell Research, Jinan, PR China; ^5^ Department of Hematology, Shandong University Second Hospital, Jinan, PR China

**Keywords:** apoptosis, BCL2, bortezomib, hTERT, telomerase

## Abstract

Bortezomib inhibits the ubiquitin/proteasome pathway to achieve its anti-cancer effect and its well characterized activity is the NF-κB inhibition through which the anti-apoptotic bcl-2 expression is down-regulated and apoptosis is subsequently induced. However, the downstream molecular targets of bortezomib are still incompletely defined. Because telomere stabilization via activation of telomerase, induction of telomerase reverse transcriptase (hTERT) and appropriate expression of shelterin proteins is essential to cancer development and progression, we investigated the effect of bortezomib on telomere homeostasis/function in malignant cells. The bortezomib treatment of leukemic (HEL) and gastric cancer cells (BGC-823) led to significant inhibition of hTERT and telomerase expression, widespread dysregulation of shelterin protein expression, and telomere shortening, thereby triggering telomere dysfunction and DNA damage. hTERT over-expression attenuated bortezomib-induced telomere shortening, abnormal shelterin expression and telomere dysfunction. Importantly, bortezomib-mediated apoptosis of malignant cells was partially prevented by hTERT over-expression. Mechanistically, hTERT first robustly enhances bcl2 expression and maintains significantly high residual levels of bcl2 even in bortezomib-treated HEL cells. Second, hTERT protects against bortezomib-induced DNA damage. Our findings collectively reveal a profound impact of bortezomib on telomere homeostasis/function. Down-regulation of hTERT expression and telomere dysfunction induced by bortezomib both contribute to its cancer cell killing actions. It is evident from the present study that hTERT can confer resistance of malignant cells to bortezomib-based target cancer therapy, which may have important clinical implications.

## INTRODUCTION

The ubiquitin/proteasome system is the major proteolytic site in mammalian cells and plays essential parts in cellular homeostasis and various physiological processes. Evidence has accumulated that the aberrant proteasome-dependent proteolysis contributes to oncogenesis, and targeting the proteasome pathway is thus an attractive anti-cancer strategy. Indeed, bortezomib, as the first proteasome inhibitor for cancer therapy, has proved successful in the treatment of hematological and other malignancies [1–6]. It is well-known that bortezomib induces cell death by inhibiting the anti-apoptotic NF-κB signaling pathway whereas enhancing the expression of the pro-apoptotic factor NOXA. However, given a broad biological and physiological activity of the proteasome system, bortezomib likely has multiple targets in malignant cells that have not been explored in details. Profound insights into bortezomib-mediated anti-cancer mechanisms should be helpful for its rational application in cancer patients.

Human telomeres are nucleoprotein complexes consisting of up to 15 - 20 kb tandemly repetitive TTAGGGs and associated proteins [7–10]. Six key proteins (TRF1, TRF2, POT1, TPP1, RAP1 and TIN2) bind to telomeric DNA and form a shelterin complex [8, 10, 11]. This telomere structure is essential for maintaining genomic stability and integrity and acts as a protective cap on human chromosome ends [7–11]. Telomere length (TL) is controlled by multiple elements and one of the major players is telomerase responsible for elongating telomeric DNA sequences [7–9]. Telomerase is silent in most human differentiated cells due to the tight repression of the *hTERT* gene, which encodes the key telomerase catalytic component [7–9]. In sharp contrast, telomerase/hTERT is widely activated in human malignancies. Activation of telomerase has been shown to be an essential step during oncogenesis, thereby stabilizing telomere length and conferring transformed cells unlimited proliferation potential [7–9]. In addition to its canonical telomere-lengthening function, hTERT or telomerase has other multiple biological activities. For instance, hTERT has been observed to enhance survival, chemo-resistance, invasion and metastasis of malignant cells independently of its telomere lengthening effect [12–17].

Because hTERT/telomerase-mediated telomere stabilization plays a key role in cancer development and progression, we are interested in potential effects of bortezomib on telomere homeostasis and function. A previous study showed that bortezomib down-regulated hTERT expression and telomerase activity in subsets of multiple myeloma (MM) cells [18], however, it remains to be defined whether the observed hTERT inhibition has any functional significances. On the other hand, as hTERT is involved in chemo- and radio-resistance of malignant cells, it appears to be important to elucidate whether hTERT is capable of protecting bortezomib-mediated apoptosis. Moreover, it is currently unclear whether bortezomib affects shelterin protein expression and telomere structure, thereby impairing telomere function in malignant cells. With all these issues in mind, we sought to elucidate the effect of bortezomib on telomere homeostasis and functional consequences.

## RESULTS

### Bortezomib treatment leads to hTERT, hTER and telomerase down-regulation in malignant cells

**Figure 1:**
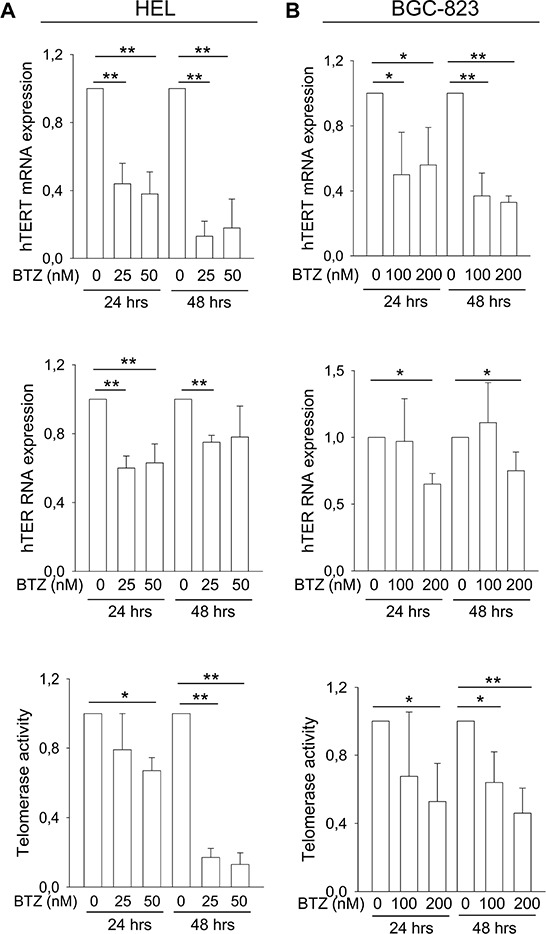
The inhibitory effect of bortezomib on hTERT and hTER expression and telomerase activity in leukemic and gastric cancer cells Cells were treated with bortezomib as indicated. hTERT and hTER transcripts were determined using qPCR and telomerase activity assessed using a PCR-ELISA kit. The levels of each target in bortezomib-treated cells were expressed as percentages of those in untreated cells. **A.** Levels of hTERT mRNA (top), hTER RNA (middle) and telomerase activity (bottom) in leukemic HEL cells treated with bortezomib. **B.** Levels of hTERT mRNA (top), hTER RNA (middle) and telomerase activity (bottom) in gastric BGC-823 cells treated with bortezomib. * and **: *P* < 0.05 and 0.01, respectively. BTZ, bortezomib.

hTERT and hTER are the core of the telomerase complex and essential to telomerase activity. hTERT expression was previously shown to be down-regulated by bortezomib in subsets of myeloma cell lines [18]. To see if this is the case in other malignant cells, we co-incubated erythroid leukemia HEL cells and gastric BGC-823 with bortezomib. Significantly diminished hTERT mRNA levels were observed in both cell lines exposed to bortezomib (Figure 1A and 1B, top panels). By 48 hours, less than 20% of the original hTERT mRNA levels were left in HEL cells and < 40% in BGC-823 cells. Bortezomib also exhibited an inhibitory effect on hTER expression to certain extent (Figure 1A and 1B, middle panels). Consistent with these changes, significant down-regulation of telomerase activity was observed in bortezomib-treated HEL and BGC-823 cells (Figure 1A and 1B, bottom panel). Of note, decreased telomerase activity developed slowly in these bortezomib-treated cells, likely due to its long half-life [19–21]. The inhibition of hTERT and telomerase by bortezomib was more efficient in HEL cells than in BGC-823 cells (Figure 1 and 1B).

### Bortezomib treatment induces widespread dysregulation of shelterin protein expression

**Figure 2:**
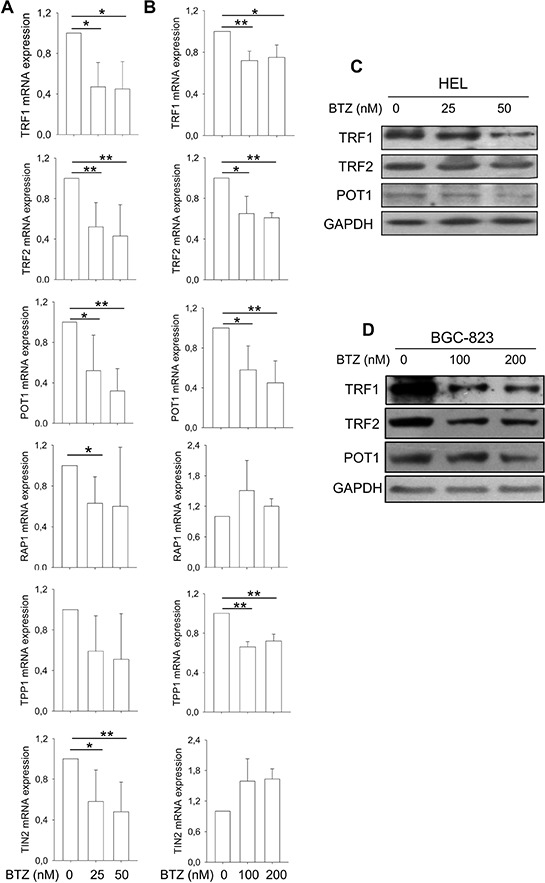
Widespread dysregulation of shelterin protein expression in bortezomib-treated HEL and BGC-823 cells **A.** and **B.** mRNA levels of shelterin factors TRF1, TRF2, TPP1, POT1, RAP1 and TIN2 in bortezomib-treated cells. Cells were treated with bortezomib for 24 hours and qPCR was used for quantitative assays. The levels of each target mRNA in bortezomib-treated cells were expressed as percentages of those in untreated cells. (A) HEL cells and (B) BGC-823 cells. **C.** and **D.** Immunoblotting assessment of TRF1, TRF2 and POT1 protein expression in bortezomib-treated cells. Same sets of cells above were analyzed for TRF1, TRF2 and POT1 protein levels and shown was representative of three independent experiments. (C) HEL cells and (D) BGC-823 cells. * and **: *P* < 0.05 and 0.01, respectively. BTZ, bortezomib.

In addition to hTERT/telomerase, shelterin proteins binding to telomere are also essential to telomere length maintenance and function [8]. We thus determined potential effects of bortezomib on shelterin protein expression. The expression of TRF1, TRF2, POT1, TPP1, RAP1 and TIN2 mRNA was significantly lower in HEL cells treated with bortezomib compared to that found in untreated cells (Figure 2A). BGC-823 cells in the presence of bortezomib exhibited different changes in shelterin mRNA levels: significant down-regulation of TRF1, TRF2, TPP1 and POT1 expression while enhanced expression of TIN2 and RAP1 (Figure 2A). We further examined TRF1, TRF2 and POT1 expression at protein levels using immunoblotting. As shown in Figure 2B, the abundance of TRF1, TRF2 and POT1 proteins was significantly decreased in bortezomib-treated HEL and BGC-823 cells, highly consistent with declines in their mRNA expression.

### Bortezomib treatment leads to telomere shortening and telomere dysfunction

**Figure 3:**
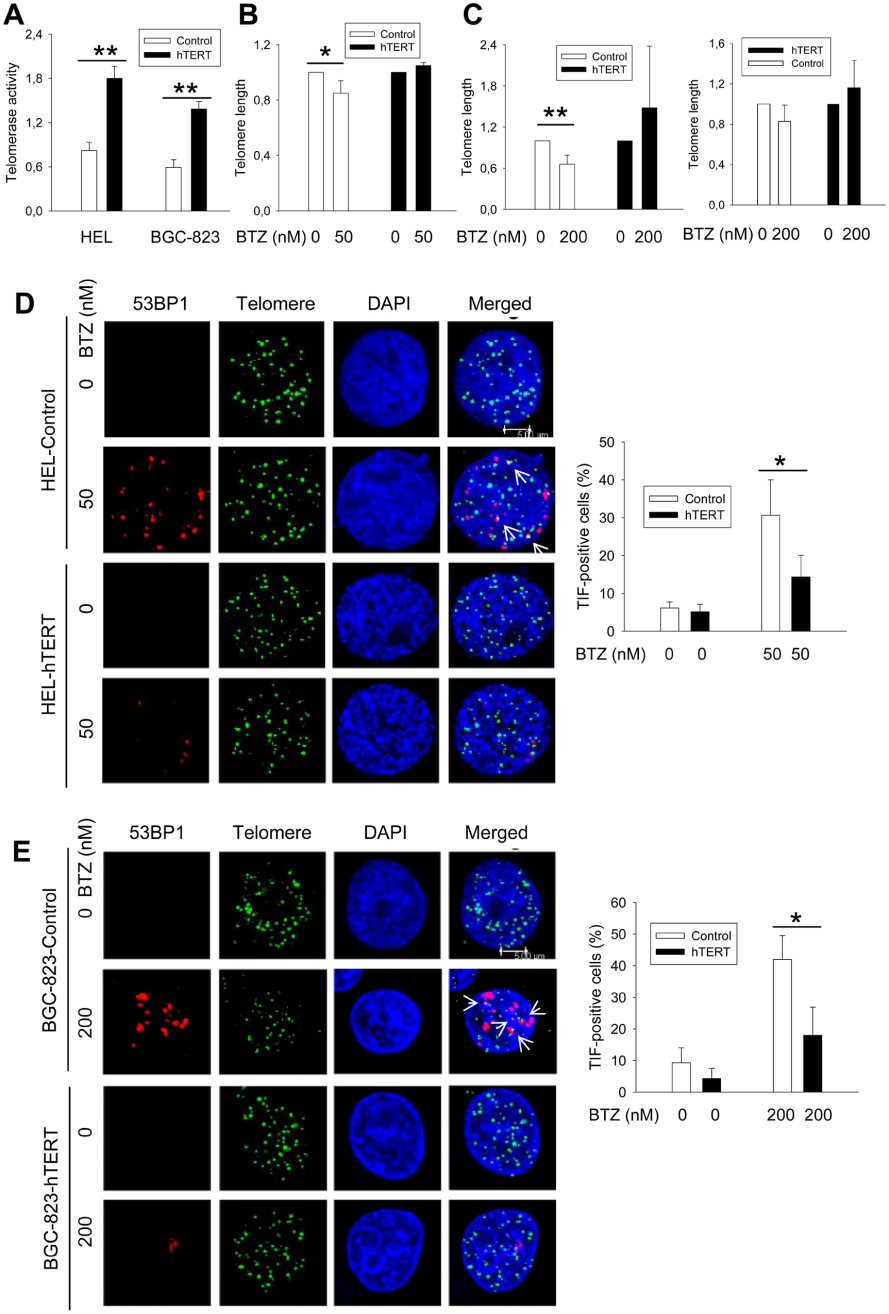
Telomere shortening and dysfunction in HEL and BGC-823 cells treated with bortezomib, which was attenuated by hTERT over-expression **A.** Enhanced telomerase activity in HEL and BGC-823 cells expressing ectopic hTERT. Cells were infected with lenti-viral or retro-viral hTERT-encoded vectors, and selected using puromycin. Telomerase activity was assessed using a telomerase PCR-ELISA kit. **B.** and **C.** Telomere shortening in bortezomib-treated control cells but not in hTERT-over-expressed cells. Cells were treated with bortezomib for 24 hours and FLOW-FISH and/or qPCR was performed to determine telomere length in these cells. B and C left panel: FLOW-FISH results; and C right panel: qPCR results. Telomere length in bortezomib-treated cells was expressed as percentages of that in untreated cells. **D.** and **E.** Telomere dysfunction in bortezomib-treated cells was inhibited by hTERT over-expression. Control and hTERT-over-expressed cells were treated with bortezomib for 24 hours and then analyzed for co-localization of 53BP1 foci and telomere signals (TIF) using immuno-FISH. Cells were counted for TIF and the percentage of positive cells (> 2 foci/cell) calculated (right panel). Shown in (D) and (E) left panels is representative of three independent experiments. * and **: *P* < 0.05 and 0.01, respectively. BTZ, bortezomib.

Having observed widespread effects of bortezomib on the expression of hTERT and shelterin proteins, we then wanted to determine whether telomere length and function was affected in bortezomib-treated cells. Flow-FISH analyses revealed that a significant telomere shortening did occur in HEL cells (means ± SD, 1.0 ± 0.0 vs 0.80 ± 0.09 for control and treated cells, respectively, *P* = 0.046) and BGC-823 cells (1.0 ± 0.0 vs 0.66 ± 0.13 for control and treated cells, respectively, *P* = 0.0016) in the presence of bortezomib (Figure 3B and 3C). Moreover, we further assessed telomere length in BGC-823 cells using quantitative PCR (qPCR), and telomere shortening was similarly observed in bortezomib-treated cells (1.0 ± 0.0 vs 0.826 ± 0.166 for control and treated cells, respectively, *P* = 0.08), although the difference was close to but did not reach a statistically significant levels (Figure 3C, right panel). To detect telomere dysfunction, we performed immuno-FISH to examine the presence of dysfunctional telomere-induced foci (TIF), or co-localization of 53BP1 foci with telomere signals. Telomeres, revealed as green signals, were readily detectable in both untreated and bortezomib-treated HEL and BGC-823 cells, whereas red 53BP1 foci only occurred in the treated cells. The merged image demonstrated that a fraction of 53BP1 foci were localized at telomeres in cells exposed to bortezomib (Figure 3D and 3E) (TIF-positive HEL cells: 6.17% ± 1.61 vs 30.67% ± 5.86 for untreated and treated cells, respectively, *P* = 0.002; TIF-positive BGC-823 cells: 6.33% ± 4.73 vs 42% ± 7.55 for untreated and treated cells, respectively, *P* = 0.003).

### hTERT over-expression inhibits bortezomib-mediated telomere dysfunction

Given the above results, we wanted to determine whether the ectopic expression of hTERT/telomerase is capable of preventing telomere dysfunction induced by bortezomib. HEL and BGC-823 cells were infected with hTERT lenti-viral and retro-viral vectors, respectively, to make hTERT-over-expressed variants, and they were then treated with bortezomib. Cells expressing ectopic hTERT contain approximately the double amount of telomerase activity compared with their empty vector-infected counterparts (Figure 3A). Upon their exposure to bortezomib, HEL-hTERT and BGC-823-hTERT cells exhibited significantly fewer TIF than did empty vector-infected cells (Figure 3. TIF positivity: 30.67% ± 5.8 vs 14.33% ± 5.77 for control and HEL-hTERT cells with bortezomib, respectively, *P* = 0.026; 42.00% ± 7.55 vs 18.00% ± 8.89 for control and BGC-823-hTERT cells with bortezomib, respectively, *P* = 0.023).

### hTERT over-expression attenuates bortezomib-induced down-regulation of TRF1, TRF2, TPP1 and RAP1 expression

**Figure 4:**
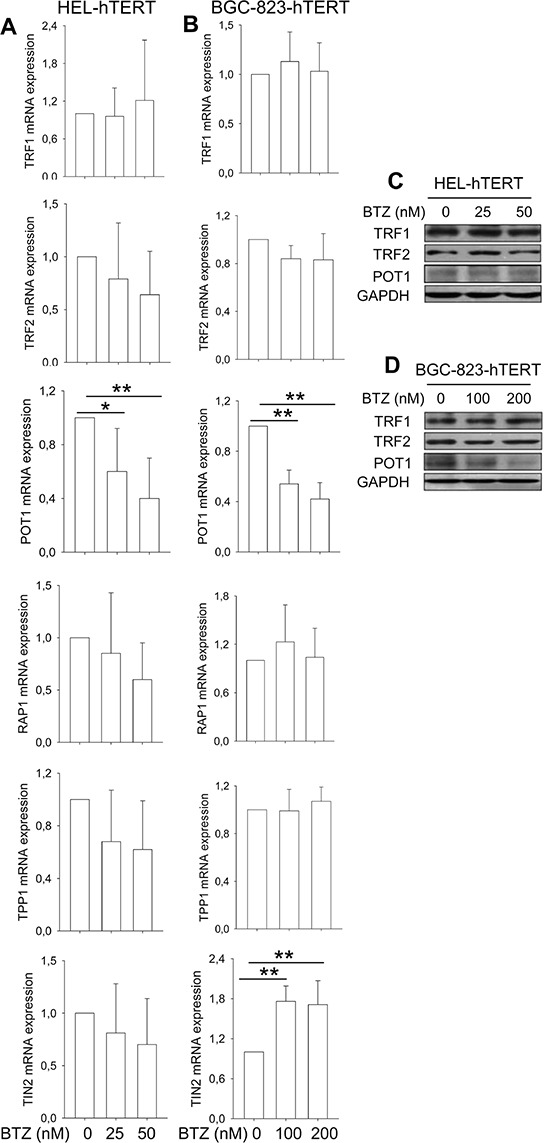
Attenuation of bortezomib-induced shelterin protein dysregulation by hTERT over-expression **A.** and **B.** Cells expressing ectopic hTERT were treated with bortezomib for 24 hours and mRNA levels of shelterin proteins then analyzed using qPCR. The levels of each target mRNA in bortezomib-treated cells were expressed as percentages of those in untreated cells. (A) HEL-hTERT cells and (B) BGC-823-hTERT cells. **C.** and **D.** Immunoblotting assessment of TRF1, TRF2 and POT1 protein expression in bortezomib-treated cells. Same sets of cells above were analyzed for TRF1, TRF2 and POT1 protein levels and shown was representative of three independent experiments. (C) HEL-hTERT cells and (D) BGC-823-hTERT cells.* and **: *P* < 0.05 and 0.01, respectively. BTZ, bortezomib.

We further sought to determine whether hTERT over-expression was capable of antagonizing bortezomib-induced dysregulation of shelterin expression, too. In HEL-hTERT and BGC-823-hTERT cells treated with bortezomib, POT1 was the only shelterin protein that was still significantly down-regulated, while TRF1 and TRF2 expression was stabilized (Figure 4A–4D), differing from that seen in these same cells without ectopic hTERT (Figure 2A–2D). RAP1 and TPP1 mRNA expression remained largely the same in cells with and without bortezomib exposure, whereas increased TIN2 transcripts occurred in treated BGC-823-hTERT cells (Figure 4).

### Bortezomib-induced cell death/apoptosis is attenuated by over-expression of hTERT

**Figure 5:**
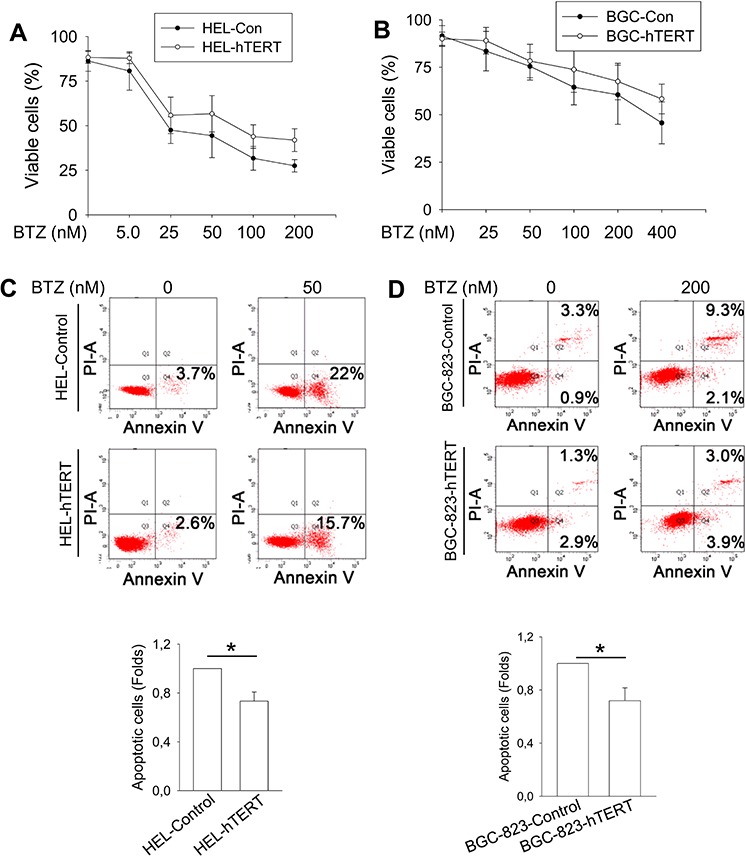
Attenuation of bortezomib-induced apoptosis by hTERT over-expression in HEL and BGC-823 cells **A.** Increased viability of BTZ-treated HEL cells expressing ectopic hTERT. Control and hTERT-over-expressed cells were treated with bortezomib for 24 hours and then counted for viable cells using trypan blue exclusion test. **B.** Increased viability of bortezomib-treated BGC-823 cells expressing ectopic hTERT. **C.** and **D.** Reduced apoptosis of BTZ-treated HEL and BGC-823 cells expressing ectopic hTERT. The same sets of cells above were stained with Annexin V and then determined for apoptotic cells using Flow cytometry. Top: Shown is representative of four independent experiments. Bottom: Apoptosis of BTZ-treated control cells was set as 1, and differences (fold changes) in BTZ-treated hTERT-cells were calculated. * and **: *P* < 0.05 and 0.01, respectively. BTZ, bortezomib.

It is well known that bortezomib treatment induces apoptotic death of cancer cells [2]. To determine the functional significance of hTERT down-regulation by bortezomib, we asked whether hTERT protects malignant cells from apoptosis induced by bortezomib. Towards this end, we treated control- and hTERT-expressed HEL and BGC-823 cells with bortezomib at different concentrations (Figure 5A and 5B). As expected, all cells incubated with bortezomib exhibited a lower viability in a dose-dependent manner (Figure 5A and 5B), however, more hTERT-over-expressed cells survived bortezomib treatment. We further performed apoptosis analysis using Annexin V staining, and observed that a significantly higher fraction of control HEL and BGC-823 cells underwent apoptosis than did hTERT-cells (Figure 5C and 5D).

### DNA damage induced by bortezomib is diminished in hTERT-over-expressed cells

**Figure 6:**
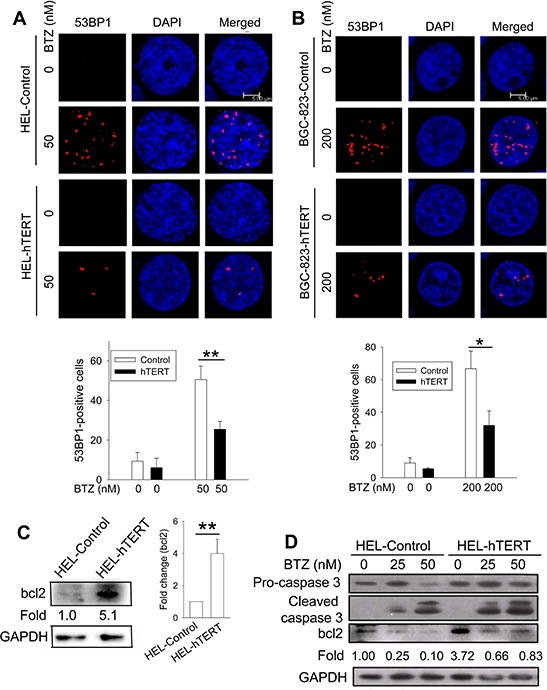
Reduced DNA damage and/or bcl-2 up-regulation by hTERT over-expression in bortezomib-treated cells **A.** and **B.** Reduced 53BP1 foci by hTERT over-expression. Immuno-fluorescence analyses were performed to detect the presence of 53BP1 foci in both control and hTERT-over-expressed cells treated with bortezomib and percentages of positive cells calculated. (A) HEL-control and HEL-hTERT cells. Top panels: representative of 53BP1 foci in HEL cells and their hTERT-over-expressed counterparts with and without bortezomib. Bottom panel: 53BP1 foci-positive cells in each group based on 4 independent experiments. (B) BGC-823-control and BGC-823-hTERT cells. * and **: *P* < 0.05 and 0.01, respectively. **C.** Bcl-2 up-regulation in HEL-hTERT cells. Left panel: Immunoblotting was performed to analyze bcl2 protein expression. Changes in folds were based on the bcl2 signal densities. Right panel: bcl2 expression levels in HEL-control and hTERT cells from four independent experiments. **D.** Bcl2 down-regulation and caspase 3 activation in bortezomib-treated HEL cells. Control and hTERT-over-expressed HEL cells were treated with bortezomib for 24 hours and then analyzed for bcl-2 expression and caspase 3 cleavage using Western blot. Bcl2 levels were calculated based on the scanned bcl2 signal densities in the image. Shown is representative of three independent experiments. BTZ, bortezomib.

Because DNA damage induced by bortezomib is required for its anti-cancer activity [22–25], we wanted to determine whether hTERT protection against apoptosis is associated with altered DNA damage in bortezomib-treated cells. For this purpose, we compared the formation of 53BP1 foci between control and hTERT cells in the presence of bortezomib. Bortezomib treatment led to 50% and 67% of 53BP1 foci-positive cells in control HEL and BGC-823 cells, respectively, while positive ones were only 25% and 32% in their hTERT-over-expressing counterparts, respectively, and percentages were significantly lower (HEL vs HEL-hTERT and BGC-823 vs BGC-823-hTERT, *P* = 0.005 and 0.01, respectively) (Figure 6A). Moreover, the number of 53BP1 foci in positive cells with ectopic hTERT was much fewer than those in positive cells containing no ectopic hTERT (Figure 6A).

### Bcl2 is robustly up-regulated by hTERT over-expression in HEL cells

Caspase 3 activation/cleavage and bcl-2 down-regulation via the NK-kB signalling inhibition is observed in bortezomib-treated MM and other malignant cells [2]. To see whether this is the case in HEL and BGC-823 cells, we incubated them with bortezomib and then analyzed caspase 3 cleavage and bcl-2 levels. Caspase 3 was undetectable in BGC-823 cells (data not shown) and we therefore only focused on HEL and HEL-hTERT cells. The immunoblotting results showed highly increased bcl-2 expression (more than 4 fold increase on average) in HEL-hTERT cells compared to their control counterparts (Figure 6C). As expected, bortezomib treatment led to bcl-2 down-regulation and caspase 3 cleavage in both control HEL and HEL-hTERT cells, however, remaining bcl2 in bortezomib-treated HEL-hTERT cells was still 80% of that in untreated control HEL cells (Figure 6D).

## DISCUSSION

Bortezomib exerts its anti-cancer effect by inhibiting the ubiquitin/proteasome pathway, however, the exact mechanisms remain incompletely understood [2]. In the present study, we show a significant impact of bortezomib on telomere homeostasis and function, which may be associated with bortezomib-mediated cancer cell killing activity.

Weise et al recently reported that bortezomib down-regulated telomerase activity accompanied by a decline in hTERT expression in subsets of MM cell lines [18]. The inhibition of hTERT expression occurred at both transcriptional and post-transcriptional levels. We similarly found a significant decline in hTERT mRNA and telomerase activity in bortezomib-treated gastric cancer and leukemic cells. These results suggest that bortezomib-mediated down-regulation of hTERT expression is not limited to MM cells. However, the question is whether such effects of bortezomib have any functional consequences. To address this, we first determined telomere length and function in bortezomib-treated cells, and shortening of telomere length coupled with telomere dysfunction did occur in these cells. To further define whether there is a causal relationship between hTERT decrease and telomere shortening/dysfunction, we then over-expressed hTERT in cells and these cells displayed no telomere shortening and significantly fewer 53BP1 foci at telomeres in response to bortezomib. More importantly, hTERT over-expression protected cells from apoptosis induced by bortezomib. Taken together, bortezomib inhibition of hTERT expression contributes to telomere dysfunction and cellular apoptosis.

The maintenance of telomere length and function depends on not only hTERT/telomerase, but also appropriate levels of shelterin protein expression [8, 11]. We observed consistent declines in expression of TRF1, TRF2, TPP1 and POT1 mRNAs in both HEL and BGC-823 cells, although changes in RAP1 and TIN2 expression differed between two cell lines. Conceivably, such widespread dysregulation of shelterin proteins might significantly disrupt their expression balance and highly organized interaction with telomeres, thereby affecting telomere structure and function. Currently the mechanisms underlying this bortezomib action remain elusive. Both mRNA and protein levels of shelterin factors were concordantly altered in bortezomib-treated cells, which indicates an indirect effect of bortezomib. Likely, bortezomib targets certain transcription factors or has epigenetic effects on shelterin gene expression. On the other hand, we unexpectedly found that hTERT over-expression was capable of attenuating abnormal shelterin expression mediated by bortezomib. It is thus plausible that the diminished hTERT expression contributes to the dysregulation of shelterin proteins in bortezomib-treated cells.

Recent studies unveil multiple biological activities of telomerase or hTERT in addition to its telomere lengthening function. For instance, hTERT is capable of facilitating the recruitment of DNA repair factors to sites of double-stranded breaks [26], while depletion of hTERT leads to enhanced cell radio-sensitivity, and diminished capacity for DNA repair [27]. In accordance with these reports, we observed that hTERT over-expression attenuated DNA damage in bortezomib-treated malignant cells. As DNA damage induced by bortezomib is required for its anti-cancer activity [22–25], it is not surprising that cell death was attenuated in hTERT over-expressed cells when exposed to bortezomib, likely due to decreased DNA damage mediated by hTERT. To our knowledge, this is the first report showing that hTERT confers cancer cells resistance to bortezomib. Thus, hTERT is capable of mediating resistance to not only conventional anti-cancer approaches, but also targeted cancer therapy, which might have broad implications in cancer therapeutics.

It has been well characterized that bortezomib inhibits the anti-apoptotic NF-κB signaling pathway by repressing its target gene expression (such as bcl-2 and others) [2]. Moreover, in this process, the activation of caspase 3 occurs, which further down-regulates bcl-2 via cleavage [2, 28, 29]. We did observe these alterations in HEL cells exposed to bortezomib. Intriguingly, a robust up-regulation of bcl2 expression was seen in HEL-hTERT cells, which might be one of important mechanisms behind hTERT protection against apoptosis by bortezomib. hTERT was recently shown to interact with NF-κB signaling and thereby promote NF-κB target expression [12, 15]. It will be interesting to determine whether hTERT and NF-κB collaboratively stimulate bcl2 expression in HEL-hTERT cells. In addition, hTERT was also observed to be involved in apoptosis by interfering bcl2 expression and function in breast cancer cells [30], which suggests broad interplay between bcl2 and hTERT in regulating cancer cell survival.

In summary, we demonstrate that bortezomib induces down-regulation of hTERT/telomerase and broad dysregulation of shelterin protein expression, thereby disrupting telomere stabilization and leading to telomere dysfunction in malignant cells. Moreover, hTERT over-expression protects from apoptotic cell death mediated by bortezomib. Collectively, the telomerase/telomere system is an important target of bortezomib. We thus suggest that future clinical trials designed to evaluate the efficacy of bortezomib include telomerase/telomere function inhibition as one indicator of response.

## MATERIALS AND METHODS

### Cells, cell culture and treatment

The erythroid leukemia cell line HEL and the gastric cancer cell line BGC-823 were used in the present study. Cells were grown in 10% foetal calf serum-containing RPMI-1640 with addition of 2 mM L-glutamine and antibiotics (50 mg/mL penicillin, and 50 mg/mL streptomycin) in a humid atmosphere at 37°C/5% CO_2_. Bortezomib was bought from Selleck Chemicals (Houston, TX, USA) and exponentially growing cells were incubated with bortezomib at different concentrations for various time periods. Cells were counted for numbers and viability using trypan blue exclusion test.

### The hTERT lenti-viral vector and cell infection

A lenti-III-HA-GFP-hTERT vector was constructed and a Lenti-BMN-GFP vector (a gift from Rudbeck Laboratory, Department of Immunology, Genetics and Pathology of Uppsala University) was used as control [19]. The lentiviral vector was packaged in 293FT cells and supernatant collected to infect HEL cells. BGC-823 cells were infected with pBABE retroviral control and hTERT vectors as described [13]. The cells were selected using puromycin.

### RNA Extraction, reverse transcription and qPCR

Total cellular RNA was extracted using Trizol kits according to the manufacture's instruction (Life Technology, Paisley, Scotland, UK). cDNA was synthesized using RevertAid First Strand cDNA Synthesis kits (Thermo Scientific, Shanghai, China) and M-MLV reverse transcriptase. qPCR was carried out in an ABI7700 sequence detector (Applied Biosystems, Foster City, CA, USA) using SYBR Green kit (Applied Biosystems, Foster City, CA) and the primer pairs specific to hTERT, hTER, TRF1, TRF2, TPP1, POT1, RAP1 and TIN2 as described [31] (Table 1). GAPDH (Table 1) was PCR-amplified as an internal control. Levels of each mRNA were calculated based on the threshold values and normalization of GAPDH expression.

**Table 1:** The primer sequences for reverse transcriptase qPCR and telomere length assessment

	PCR primer sequences for mRNA quantification	
Target	Forward	Reverse
TRF1	5′-GCTGTTTGTATGGAAAATGGC-3′	5′-CCGCTGCCTTCATTAGAAAG-3′
TRF2	5′-GACCTTCCAGCAGAAGATGCT-3′	5′-GTTGGAGGATTCCGTAGCTG-3′
TPP1	5′-CCCGCAGAGTTCTATCTCCA-3′	5′-GGACAGTGATAGGCCTGCAT-3′
TIN2	5′-GGAGTTTCTGCGATCTCTGC-3′	5′-GATCCCGCACTATAGGTCCA-3
POT1	5′-TGGGTATTGTACCCCTCCAA-3′	5′-GATGAAGCATTCCAACCACGG-3′
RAP1	5′-CGGGGAACCACAGAATAAGA-3′	5′-CTCAGGTGTGGGTGGATCAT-3′
hTERT	5′-CGGAAGAGTGTCTGGAGCAA-3′	5′-GGATGAAGCGGAGTCTGGA-3′
GAPDH	5′-AAAGGGCCCTGACAACTCTT-3′	5′-GGTGGTCCAGGGGTCTTACT-3′
hTERC	5′-TCTAACCCTAACTGAGAAGGGCGTAG-3′ (forward)	
	5′-GTTTGCTCTAGAATGAACGGTGGAAG-3′ (reverse)	
	**Primer sequences for telomere length analyses**	
Tel1b	5′-CGGTTTGTTTGGGTTTGGGT-TTGGGTTTGGGTTTGGGTT-3′	
Tel2b	5′-GGCTTGCCTTACCCTTACCCTTACCC-TTACCCTTACCCT-3′	
HBG3	5′-TGTGCTGGCCCATCACTTTG-3′	
HBG4	5′-ACCAGCCA-CCACTTTCTGATAGG-3′	

### Immunoblotting

Total cellular proteins were extracted using RIPA lysis buffer, then subjected to sodium dodecyl sulfate-polyacrylamide gel electrophoresis and transferred to a PVDF membrane. The membranes were probed with the specific antibodies against TRF1 (Sigma-Aldrich, St. Louis, MO, USA, T1948), TFR2 (Novus, Littleton, CO, USA, #NB110–57130), POT1 (Novus, NB500–176), Caspase3 (Cell Signaling Technology, Danvers, MA, USA, #9665), or Bcl-2 (ProteinTech, Wuhan, China, #12789–1-AP) followed by anti-mouse or rabbit horseradish peroxidase-conjugated IgG and developed with the enhanced chemiluminescent method (SuperSignal West Pico Chemiluminescent Substrate, Thermo Scientific). GAPDH Santa Cruz Biotechnology, Inc., Santa Cruz, CA, USA, #sc-47724) immunoblotting was performed in parallel as a loading control.

### Telomerase activity determination

Telomerase activity was assessed using a commercial Telomerase PCR ELISA kit (Shanghai Roche Pharmaceuticals Ltd., Shanghai, PR China) as recommended by the manufacturer. For each assay, one μg of protein was used, and 23 PCR cycles were performed after the telomerase-primer elongation reaction. The PCR products were detected using ELISA color reaction and the level of telomerase activity was expressed as absorbance in arbitrary units.

### Flow cytometry analysis of apoptosis

Cells were treated with bortezomib as decribed above, and then harvested for apoptosis assay. The cells were stained with a kit from BD pharmingen using a FlowCytometer (BD). For each sample 1 × 10^6^ cells were measured. The control gate was set based on the negative control.

### Immuno-FISH

Immuno-FISH was carried out as described [32]. Briefly, cells were harvested, cytospined onto Superfrost plus slides (Thermo Scientific), fixed with 4% paraformadehyde and permeabilized with Triton PBS followed by a block step with serum free Block (DAKO, Glostrup, Denmark). The cells were first incubated with 53BP1 antibody (Bethyl Inc., Montgomery, Texas, USA) and then with Alexa 594 secondary antibody (Jackson Labs Technologies Inc., Los Gatos, CA, USA). The PNA-telomere probe (PANAGENE Inc., Daejeon, Korea) was finally added onto the slides and the results were analyzed using a confocal microscopy Leica TCS SP5 (Mannheim, Germany). Cell carrying > 2 co-localization foci is defined as having telomere dysfunction.

### Flow-FISH and qPCR for telomere length assay

Flow FISH of cells was performed according to a previous protocol by Baerlocher et al [33–35] with minor modifications. Cells from calf thymus were kindly donated from the butchery Ö-slakt AB (Värmdö, Stockholm). All experiments were made with a Gallios flow cytometer (Beckman Coulter) and analyzed using the Kaluza software (Beckman Coulter, Caguas, PR, USA). For quantification fluorescent MESF-FITC beads (Bangs Laboratories, Fishers, IN, USA) were used and the fluorescent signal was quantified using the QuickCal v.2.3 data analysis program (Bangs Laboratories). For qPCR, genomic DNA was isolated and telomere length was assessed by real-time PCR as described [30, 36]. Two ng of DNA were used for each PCR reaction. The primer sequences for human telomere (Tel 1b and Tel 2b) and β-globin (HBG3 and HBG4) were listed in Table 1: T/HBG values were determined using the formula T/S = 2^−ΔCt^, where ΔCt = average Ct_telomere_ − average Ct_β-globin_ and relative telomere length was expressed as the percentage of that in BGC-823 cells without bortezomib.

### Statistics

Student's *t*-test or Mann-Whitney U test was used to compare cell numbers, apoptotic cells, hTERT, hTERC and shelterin protein mRNA levels and telomere length between control and bortezomib-treated cells. All the tests were two-tailed and computed using SigmaStat3.1^®^ software (Systat Software, Inc., Richmond, CA, USA). *P* values of < 0.05 were regarded as statistically significant.
